# Validity and reliability of a Korean version of the Consultation and Relational Empathy (CARE) measure

**DOI:** 10.1186/s12909-022-03478-5

**Published:** 2022-05-26

**Authors:** Kye-Yeung Park, Jinho Shin, Hoon-Ki Park, Yu Mi Kim, Seon Young Hwang, Jeong-Hun Shin, Ran Heo, Soorack Ryu, Stewart W. Mercer

**Affiliations:** 1 Department of Family Medicine, Hanyang University College of Medicine, 222 Wangsimni-ro, Seongdong-gu, Seoul, South Korea; 2 Division of Cardiology, Department of Internal Medicine, Hanyang University College of Medicine, Seoul, South Korea; 3 Department of Preventive Medicine, Hanyang University College of Medicine, Seoul, South Korea; 4 School of Nursing, Hanyang University, Seoul, South Korea; 5 Division of Cardiology, Department of Internal Medicine, Hanyang University Guri Hospital, Hanyang University College of Medicine, Guri, South Korea; 6 Biostatistical Consulting and Research Lab, Medical Research Collaborating Center, Hanyang University, Seoul, South Korea; 7 Centre for Population Health Sciences, Usher Institute, College of Medicine and Veterinary Medicine, University of Edinburgh, Edinburgh, Scotland

**Keywords:** Empathy, Consultation, Translation, Factor Analysis

## Abstract

**Background:**

No validated tool is available to assess patients’ perception of physician empathy in Korea. The objective of this study was to establish a Korean version of the Consultation and Relational Empathy (CARE) measure—originally developed in English and widely used internationally—and to examine its reliability and validity.

**Methods:**

The CARE measure was translated into Korean and tested on 240 patients from one secondary care hospital and one tertiary care hospital in Korea. Internal consistency by Cronbach’s alpha, exploratory analysis, and confirmatory factor analysis were conducted to verify the 10 items of the Korean CARE measure.

**Results:**

The Korean CARE measure demonstrated high acceptability and face validity, excellent internal reliability (Cronbach’s alpha = 0.97) and moderate test-retest reliability (Pearson correlation coefficient = 0.53; Spearman correlation coefficient = 0.51). Distribution of scores showed negative skewedness. Corrected item-total correlations ranged from 0.77–0.92, indicating homogeneity. The Kaiser-Meyer-Olkin measure of sampling adequacy was 0.949, and Bartlett’s test of sphericity was good (χ2 = 3157.11, *P* < 0.001). Factor analysis yielded a single dimensional structure of physician empathy with all factor loadings exceeding 0.80 and showing excellent goodness of fit.

**Conclusion:**

This study supports the reliability and validity of the Korean CARE measure in a university hospital setting in Korea.

**Supplementary Information:**

The online version contains supplementary material available at 10.1186/s12909-022-03478-5.

## Background

Patient-centeredness is to understand each patient as a whole-person with consideration for patients’ values, needs, and preferences, as a core concept of contemporary medical care [1, 2]. Empathy is an essential component of patient-centeredness, which can be defined as the ability to understand the patient’s situation, perspective and feelings and to communicate with patients in a therapeutic way [3, 4]. Numerous studies support the positive relationships between physician empathy and a variety of health outcomes such as patient satisfaction, medication adherence, patient recovery, emotional health, and even physician’s satisfaction [4–6].

According to Hojat [3], empathy in patient care situations is a cognitive attribute that involves an ability to understand the patient’s inner experiences and perspective and a capability to communicate this understanding. It has been considered that empathy has both cognitive and affective nature [3, 7]. The ability to understand the patient’s inner experiences and feelings from the patients’ point of view can be described as cognitive domains of clinical empathy [3]. Meanwhile, the affective domain of empathy involves the capacity to enter into the experiences and feelings of the patients that often elicit emotional responses [8]. Empathic communication between physicians and patients affects patients’ adherence and motivation for treatment, ultimately leading to improved health outcome [9]. On the other hand, communication lacking empathy provokes patient resistance [10]. A patient-centered approach based on empathy is now regarded as an underpinning requirement beginning at the undergraduate level, and every physician is encouraged to reinforce empathic communication skills in their daily work [11–13].

Assessment instruments to measure physician empathy in healthcare settings have been explored and validated [14–17]. Measuring physician empathy from the patient view could allow assessment and feedback of strengths and weaknesses of physicians’ empathic behavior [18, 19], rather than physicians’ self-evaluation or third-party evaluation methods. The Consultation and Relational Empathy measure (CARE) was developed in the UK to assess patient perceptions of relational empathy and communication during consultation with physicians [17]. Since its development and validation, the CARE measure has been widely used in the UK, from workplace-based assessment to high-stakes assessment [20, 21]. It has been translated and fully validated in culturally and linguistically diverse populations such as German, Chinese, Japanese, Croatian, Swedish, and most recently, Spanish [22–28].

Korea has been striving to move toward patient-centered medical care, as healthcare authorities assess patient satisfaction at the national level. However, despite such efforts, there are no valid and reliable measures in the Korean language to assess physician empathy or patient-centeredness from the patient point of view. Establishing such an instrument in Korean language could be beneficial to improving physicians’ skills on empathic communication. Previous studies conducted in Korea on patient-physician relationship using the CARE measure further prompted us to develop a Korean version of CARE measure [29, 30]. Thus, we aimed to translate the CARE measure into Korean and to examine the validity and reliability of a Korean version of the CARE measure.

## Methods

### Translation of the CARE measure

The CARE measure has 10 items measuring physician empathy with a 5-point Likert scale ranging from ‘poor’ to ‘excellent’, as well as a ‘not applicable’ option [17]. The total scale score ranges from 10 to 50. After receiving approval by e-mail from the CARE developers for use in Korean patients, we first translated the original CARE measure into Korean. The first draft was commissioned by the Interpretation and Translation Centre of Hankuk University of Foreign Studies, and two bilingual professional interpreters went through the primary translation. A back translation of the first draft was conducted by another two independent staff members of the Interpretation and Translation Centre, and they reviewed whether there were any items that needed correction to improve translation and cultural accuracy. Next, two bilingual (English/Korean) academic staff compared the original questionnaire and the reverse translated version of the questionnaire to create the second draft. After that, a research team of six experts (two professors from the departments of family medicine (HKP) and preventive cardiology (JHS) in a secondary care hospital, two professors (RH & JS) in a tertiary care hospital, and two professors from the department of preventive medicine (YMK) and nursing (SYH) who were experienced in tool development) held an on-line meeting to compare the second draft with the original CARE measure to verify its meaning and reconfirm that it was properly translated. At the meeting, it was decided to present two versions of six conflicting questions to patients during upcoming cognitive interviews to resolve the conflicts in the second draft.

The face validity of the second draft of the Korean version was confirmed in the second expert meeting. Cognitive interviews with eight patients including four elderly people who visited the outpatient clinics and a trained research nurse were conducted using the second draft of the Korean-CARE Measure. Patients were asked to assess the readability of the translated tool, offer their opinions about any comprehension difficulties, and report which question version was easier to understand for the questions that had two different versions. During these interviews, questions that were difficult to understand were identified, and parts that needed more interpretation such as ‘choice options,’ ‘vague,’ and ‘positive versus negative’ were identified. Based on interview results, question formats and vocabulary were corrected and supplemented through comparison with the original CARE measure during the second expert meeting, which was when content validity was verified for the final draft of the Korean CARE measure [see Additional file 1].

### Data collection for validation and reliability of the Korean CARE measure

The study subjects were patients aged 20 years or older who visited one of three clinics, the one cardiology outpatient clinic at a secondary care hospital, one cardiology outpatient clinic at a tertiary care hospital, or one family medicine outpatient clinic at the same tertiary care hospital, from February to July 2021. The main diagnosis of patients consisted of angina, myocardial infarction, valvular heart disease, heart failure, arrhythmia, hypertension, hyperlipidemia, or diabetes mellitus. Those who could read and communicate in Korean and gave their written consent to participate in the study were included, while those who had been diagnosed with an organic brain disease or a psychiatric disorder and those who refused to participate in the study were excluded. Generally, structural equation modeling requires at least 10 observations per estimated parameter or a minimum sample size of 200 [31, 32]. A total of 240 patients were final subjects. A research nurse explained the purpose of the study in a one-on-one setting to the study subjects. An anonymous and a paper and pencil-version of the Korean CARE measure questions was completed by the patients after their clinical encounter. Demographic information on age and sex was collected. To verify test-retest reliability, 35 patients whose second outpatient clinic visit was scheduled 2 weeks later were selected based on consecutive convenience sampling method. Although there was no previous literature verifying test-retest reliability of the CARE measure, we assumed that the correlation coefficient of test-retest reliability was 0.5 at least, and the minimum required sample size was calculated to be 29 or more [31]. They completed the Korean CARE measure repeatedly before the second consultation.

The Institutional Review Board of Hanyang University Hospital approved this study (No.2020–09-007). The study was conducted in compliance with the Helsinki Declaration. Informed consent was submitted by all subjects when they were enrolled.

### Statistical analysis

The acceptability and face validity of the Korean version of the CARE measure were assessed by the number of ‘not applicable’ scores and missing values for each of the 10 items. Internal reliability was determined by Cronbach’s alpha, and we examined whether removal of any of the 10 items weakened Cronbach’s alpha. Homogeneity was examined by corrected item-total correlations, where a value > 0.20 was a predictor of high correlation. Test-retest reliability was determined by calculating Pearson and Spearman’s rho correlation between two measurement timepoints from 35 patients. Exploratory factor analysis (EFA) was performed to examine the internal structure of correlations for the Korean CARE measure and to determine if the items within the measure formed a distinct construct. Varimax rotation with Kaiser normalization was used as well as Kaiser criterion for retaining components with eigenvalue > 1. Confirmatory factor analysis (CFA) was conducted to examine whether the intended constructs were measured. The appropriateness of a specific CFA model was assessed according to global and local fit using the following standards: root mean squared residual (RMR, ≤0.05), standardized root mean squared residual (SRMR, ≤0.1), Tucker-Lewis index (TLI, ≥0.90), normed fit index (NFI, ≥0.90), comparative fit index (CFI, ≥0.95), incremental fit index (IFI, ≥0.90), and relative fit index (RFI, ≥0.90) [33–35]. CFA was performed using LISREL 8.52 (Scientific Software International, Inc., Lincolnwood, IL, USA), while other statistical analyses including EFA were performed using IBM SPSS version 26.0 (IBM Corp., Armonk, NY, USA).

## Results

### Collected questionnaires

A total of 240 questionnaires was collected from clinics in the departments of cardiovascular and family medicine. A total of 9 doctors, seven male doctors and two female doctors, met with 240 patients, all of whom were attending physicians in Cardiology and Family medicine. They consisted of 3 tenured professors, 2 associate professors, 3 assistant professors, and 1 clinical assistant professor. Of the 240 study subjects, 129 (53.8%) were male, and patient age ranged from 22 to 94 years with a mean age of 59.9 ± 14.3 years. Male patients had lower scores from the Korean version CARE measure than females (*P*-value< 0.02), and patients who visited the cardiovascular clinic had lower scores than those from the family medicine clinic (*P*-value< 0.01) (Table 1).

**Table 1:** Patient demographic characteristics and empathy scores according to a Korean version of the CARE Measure

	n(%)	CARE score, mean (SD)	P*
Age (years)			0.14
39 or less	21 (8.7)	47.24 (6.47)	
40–64	129 (53.8)	45.64 (7.05)	
≥ 65	90 (37.5)	47.27 (4.91)	
Sex			0.02
Male	129 (53.8)	45.59 (7.41)	
Female	111 (46.2)	47.32 (4.57)	
Department visited			0.01
Cardiovascular, tertiary	112 (46.7)	45.36 (6.97)	
Cardiovascular, secondary	30 (12.5)	45.37 (5.80)	
Family medicine	98 (40.8)	47.88 (5.35)	
Marital status			0.09
Married with living	138 (57.5)	45.58 (6.90)	
Unmarried or married without living	23 (9.6)	46.74 (6.77)	
Missing^a^	79 (32.9)	47.32 (5.09)	
Education			0.33
Elementary school or below	41 (17.1)	47.07 (5.39)	
Middle and High school	53 (22.1)	45.57 (8.19)	
College or above	32 (13.3)	44.50 (7.42)	
Missing^a^	114 (47.5)	46.79 (5.30)	
Occupation			0.07
Yes	61 (25.4)	46.11 (7.39)	
No	98 (40.8)	45.54 (6.64)	
Missing^a^	81 (33.8)	47.25 (5.04)	
Total	240 (100)	46.39 (6.31)	

*Abbreviation*: *CARE* Consultation and Relational Empathy, *SD* standard deviation

*P-values were calculated using the Mann-Whitney test or Kruskal-Wallis test

^a^Missing value of the self-administered questionnaire on demographic characteristics

Table 2 shows the response pattern for the Korean CARE measure. The average score of the CARE measure among all subjects was 46.4 ± 6.3 (range: 10–50), and 56.7% rated the maximum possible score of 50. The option ‘not applicable’ was observed for items 4,5,6,8,9 and 10, wherein these items up to 0.4% of study participants chose not applicable response. There were no missing values for any items. The measure developers allowed up to two ‘does not apply’ responses or missing values and recommend replacing these values with the average value of the remaining items.

**Table 2:** Descriptive data on response pattern to the 10 items in the Korean CARE measure

Items	Poor (%)	Fair (%)	Good (%)	Very Good (%)	Excellent (%)	Not applicable (%)	Total (%)
1. Making patient feel at ease	3 (1.3)	4 (1.7)	2 (0.8)	44 (18.3)	187 (77.9)	0 (0)	240 (100)
2. Letting patient tell their ‘story’	3 (1.3)	3 (1.3)	3 (1.3)	57 (23.8)	174 (72.5)	0 (0)	240 (100)
3. Really listening	2 (0.8)	3 (1.3)	3 (1.3)	56 (23.3)	176 (73.3)	0 (0)	240 (100)
4. Being interested in patient as whole person	3 (1.3)	4 (1.7)	3 (1.3)	60 (25.0)	169 (70.4)	1 (0.4)	240 (100)
5. Fully understanding patient’s concerns	2 (0.8)	5 (2.1)	3 (1.3)	57 (23.8)	172 (71.7)	1 (0.4)	240 (100)
6. Showing care and compassion	2 (0.8)	7 (2.9)	3 (1.3)	57 (23.8)	170 (70.8)	1 (0.4)	240 (100)
7. Being positive	2 (0.8)	3 (1.3)	5 (2.1)	58 (24.2)	172 (71.7)	0 (0)	240 (100)
8. Explaining things clearly	2 (0.8)	3 (1.3)	4 (1.7)	54 (22.5)	176 (73.3)	1 (0.4)	240 (100)
9. Helping patient to take control	2 (0.8)	9 (3.8)	5 (2.1)	61 (25.4)	162 (67.5)	1 (0.4)	240 (100)
10. Making a plan of action with patient	2 (0.8)	5 (2.1)	5 (2.1)	61 (25.4)	166 (69.2)	1 (0.4)	240 (100)

*Abbreviation*: *CARE* Consultation and Relational Empathy

### Analysis of the reliability of the CARE measure

The test-retest reliability of the 10 items was 0.53 for Pearson correlation coefficient and 0.51 for Spearman correlation coefficient (*P* < 0.001; Table 3). Cronbach’s alpha for the scale was 0.97, which indicated excellent internal reliability. Cronbach’s alpha would not be improved by eliminating any of the item. Corrected item-total correlations were high and ranged from 0.77 for item one to 0.92 for item three suggesting prominent homogeneity (Table 4). Not shown in table, Cronbach’s alpha according to age was 0.98 in patients under 50 years old and 0.97 in those aged 50 years or above; according to sex, 0.98 in male patients and 0.95 in female patients; according to department, 0.97 in cardiovascular department of tertiary care hospital, 0.98 in cardiovascular department of secondary care hospital, and 0.98 in family medicine department.

**Table 3:** Test-retest reliability for the Korean CARE measure

Items	First (*n* = 35)		Second (*n* = 35)		Second - First (*n* = 35)		P by signed rank test	Correlation coefficient	
	mean ± SD	median (range)	mean ± SD	median (range)	mean ± SD	median (range)		Pearson	Spearman
Item 1	4.46 ± 1.07	5 (1, 5)	4.51 ± 0.78	5 (2, 5)	0.06 ± 1.08	0 (−3, 4)	0.816	0.345	0.262
Item 2	4.37 ± 1.09	5 (1, 5)	4.51 ± 0.85	5 (2, 5)	0.14 ± 1.14	0 (− 3, 4)	0.446	0.327	0.275
Item 3	4.54 ± 0.89	5 (1, 5)	4.51 ± 0.85	5 (2, 5)	−0.03 ± 0.86	0 (−3, 2)	0.973	0.515	0.396
Item 4	4.51 ± 0.89	5 (1, 5)	4.34 ± 0.97	5 (2, 5)	−0.17 ± 0.89	0 (−3, 1)	0.273	0.542	0.446
Item 5	4.46 ± 0.92	5 (1, 5)	4.40 ± 0.91	5 (2, 5)	−0.06 ± 0.94	0 (−3, 2)	0.804	0.476	0.406
Item 6	4.37 ± 0.97	5 (1, 5)	4.37 ± 0.94	5 (2, 5)	0.00 ± 0.91	0 (−3, 2)	0.894	0.551	0.523
Item 7	4.49 ± 0.85	5 (1, 5)	4.46 ± 0.82	5 (2, 5)	−0.03 ± 0.79	0 (−3, 1)	1.000	0.558	0.490
Item 8	4.49 ± 0.82	5 (1, 5)	4.51 ± 0.82	5 (2, 5)	0.03 ± 0.82	0 (−3, 1)	0.675	0.495	0.397
Item 9	4.26 ± 1.09	5 (1, 5)	4.40 ± 0.91	5 (2, 5)	0.14 ± 1.06	0 (−3, 2)	0.364	0.453	0.426
Item 10	4.43 ± 0.88	5 (1, 5)	4.40 ± 0.95	5 (2, 5)	−0.03 ± 0.95	0 (−3, 2)	0.896	0.457	0.424
Total	44.37 ± 8.69	50 (10,50)	44.43 ± 8.43	50 (20,50)	0.06 ± 8.33	0 (−30,16)	0.653	0.528	0.513
Cronbach’s alpha	0.978		0.989		0.966				

*Abbreviation*: *CARE* Consultation and Relational Empathy, *SD* standard deviation

**Table 4:** Reliability, homogeneity, exploratory and confirmatory factor loadings for the Korean CARE measure

Items	Scale mean if item deleted	Corrected item-total correlation	Cronbach’s α if item deleted	EFA Factor loading	CFA Factor loading
1. Making patient feel at ease	32.71	0.765	0.974	0.806	0.769
2. Letting patient tell their ‘story’	32.76	0.872	0.970	0.897	0.871
3. Really listening	32.74	0.916	0.969	0.934	0.925
4. Being interested in patient as whole person	32.78	0.822	0.972	0.855	0.832
5. Fully understanding patient’s concerns	32.77	0.907	0.969	0.927	0.925
6. Showing care and compassion	32.79	0.901	0.969	0.923	0.923
7. Being positive	32.77	0.903	0.969	0.924	0.920
8. Explaining things clearly	32.74	0.897	0.969	0.919	0.917
9. Helping patient to take control	32.85	0.870	0.970	0.896	0.887
10. Making a plan of action with patient	32.80	0.885	0.970	0.907	0.895
Eigen value				8.090	
% of variance				80.90%	
Kaiser-Meyer-Olkin test				0.949	
Bartlett’s test of sphericity test					
Bartlett’s χ^2^				3157.11	
Degree of freedom				45	
Significance (P)				< 0.001	

*Abbreviation*: *CARE* Consultation and Relational Empathy, *EFA* exploratory factor analysis, CFA, confirmatory factor analysis. Total Cronbach’s alpha 0.97

Goodness-of-fit indices: root mean squared residual (RMR) = 0.034; standardized root mean squared residual (SRMR) = 0.034; Tucker-Lewis index (TLI) = 0.957; normed fit index (NFI) = 0.961; comparative fit index (CFI) = 0.966; incremental fit index (IFI) = 0.966; relative fit index (RFI) = 0.950. Cut-offs used to indicate goodness of fit: RMR ≤ 0.05; SRMR≤0.1; TLI ≥ 0.90; NFI ≥ 0.90; CFI ≥ 0.95; IFI ≥ 0.90; RFI ≥ 0.90

### Analysis of the validity of the Korean CARE measure

Table 4 also shows the results from EFA and CFA. The Kaiser-Meyer-Olkin measure of 0.949 and Bartlett’s test of sphericity (χ2 = 3157.11, *P* < 0.001) confirmed the validity and sampling adequacy of our data. Using a principal component analysis, one factor was retained with 80.90% of the variance. All 10 items loaded significantly on this single factor. No items were discarded as factor loadings, and all exceeded 0.80. Factor analysis implied a one-dimensional structure with factor loadings between 0.81 and 0.93. In analysis of the validity of the Korean CARE measure with CFA, this one-factor model met the criteria of excellent goodness of fit (RMR = 0.03; SRMR = 0.03; TLI = 0.96; NFI = 0.96; CFI = 0.97; IFI = 0.97; RFI = 0.95). The null hypothesis of the chi-square test was rejected (χ2 = 137.72; *P* < 0.001), suggesting an adequate fit of the data with the one-factor model.

## Discussion

In this study, we translated the original CARE measure into Korean and examined its validity and internal consistency in a university hospital setting in Korea. High acceptability and face validity were observed as small number of ‘not applicable’ responses and missing values. An excellent internal consistency was indicated by a high Cronbach’s alpha. Factor analysis suggested that all 10 items in the Korean CARE measure assessed a single dimension of physician empathy and showed an excellent model of fit, demonstrating similar findings to that of the original English language version [17] and previous translations [23–26, 28, 36].

Our results indicated that the Korean CARE measure, the first measurement tool to evaluate physician empathy from the patient’ perspective, has adequate psychometric properties. The original CARE developers defined physician empathy multidimensionally with four components: emotive, moral, cognitive, and behavioral [37]. They described that the CARE measure captures all components of empathy [17]. As for methods to measure physician empathy translated and validated in Korean, the Jefferson Scale of Physician Empathy (JSPE) [38] and the Interpersonal Reactivity Index (IRI) [39] are currently available, and are the two most common instruments worldwide. The Korean version of JSPE consists of 18 items and seven scales to self-assess empathy of physicians or medical students in the context of patient-physician relationships [38]. The IRI is a self-assessment instrument to assess one’s own empathic abilities with 28 items [39]. Compared to JSPE and IRI, the Korean CARE measure can be an effective feedback tool to self-reflect on the strengths and weaknesses of physicians’ empathic behavior from the patient perceptive as well as being a timesaving tool.

Alongside its satisfying psychometric properties, several other aspects of the score characteristics of the Korean CARE measure should be addressed. The distribution of scores showed a markedly negative skewness that is similar to those found in some earlier works [25–28]. The mean score of 46.4 in this study was higher compared with the original English-version CARE measure of 42.4 and other samples from different ethnicities or countries (Hispanic, 42.22 ± 8.25; Swedish, 41.66 ± 8.48; India 43.80 ± 5.36; Japanese, 38.41 ± 8.6; Chinese, 31.46 ± 8.70) [23, 24, 26–28]. Additionally, maximal respondent rate in our study was prominent, as seen in the Swedish sample where 40 to 50% scored the maximum score and the Croatian sample where 86.9% marked the good or very good for almost all items [25, 26]. Being asked to fill out a questionnaire right after clinical encounters with their usual physicians could have made it difficult for the patients to rate the physician below average. The lack of atmosphere for patient-centered whole-person care and a possibly short consultation time in a busy medical environment in Korea might prevent patients from conducting a detailed evaluation of their physician’s empathy. Additionally, a tertiary care setting, where frequent procedures requiring high levels of expertise are performed or where longstanding patient-physician relationships occur, might have affected patient’ perception of physician empathy, leading to a halo effect.

As mentioned in previous studies [23, 24, 27], considerable time and effort were required to establish an accurate and culturally appropriate translation of the English CARE measure into Korean. With our results as preliminary evidence, further studies can be performed to expand the psychometric evidence for the Korean CARE measure. One could examine how the Korean CARE measure correlates with patient outcome or patient satisfaction and how direct feedback using the measure affects and improves physicians’ empathic skills. Other aspects of validity such as criterion-validity of the Korean CARE measure should also be tested. Additional research could be undertaken to examine the correlations between consultation characteristics, patient characteristics, and CARE scores in the Korean setting, although it has been reported that the duration of consultation and how well the patient knew the doctor had a small influence on CARE measure score [40–42]. It would also be worthwhile to perform in-depth psychometric analyses to examine key metric properties of the Korean CARE measure, as previously examined [43, 44].

There are several limitations in our study. One limitation is the selective patient sample and the single setting of the secondary/tertiary hospital. Larger studies in diverse settings are needed to confirm the utility of the Korean CARE measure. Also, because information on response rate was unavailable, it was not possible to evaluate the characteristics of participating patients against those who refused to participate, making it difficult to confirm the representativeness of the sample. Lastly, the large number of missing values for some socioeconomic variables and consultation characteristics (time and first visit/revisit, etc.) limited further analysis.

## Conclusion

We established the Korean CARE measure and tested its psychometric properties in a secondary/tertiary hospital in Korea. The results suggest that the Korean CARE measure is valid and reliable in our sample. This is the first important milestone measuring patient perception of physician empathy in a Korean medical environment. Further in-depth analysis that confirms the unidimensionality of the scale and identification of moderating variables is warranted to improve the theoretical foundation of the Korean CARE measure.

## Supplementary Information

**Additional file 1.**

## Data Availability

The datasets used and/or analysed during the current study are available from the corresponding author on reasonable request.

## Abbreviations

CARE: Consultation and Relational Empathy
CFA: Confirmatory factor analysis
EFA: Exploratory factor analysis
RMR: Root mean squared residual
SRMR: Standardized root mean squared residual
TLI: Tucker-Lewis index
NFI: Normed fit index
CFI: Comparative fit index
IFI: Incremental fit index
RFI: Relative fit index
JSPE: Jefferson Scale of Physician Empathy
IRI: Interpersonal Reactivity Index
SD: Standard deviation
