# Supplementary material for: Validity and reliability of a Korean version of the Consultation and Relational Empathy (CARE) measure
Source: BMC Med Educ. 2022 May 26;22:403. doi: 10.1186/s12909-022-03478-5 (PMC9134586; doi:10.1186/s12909-022-03478-5)
Supplement: Supplementary file 1 — Additional file 1. [file 12909_2022_3478_MOESM1_ESM.docx]

Additional file 1. The Korean CARE measure

| **CARE 조사** | | | | | | |
| --- | --- | --- | --- | --- | --- | --- |
| **오늘 진료에 대해 아래 문항을 평가해주세요.**  각 문항별로 하나의 답을 선택해주세요. ☑ 모든 항목에 답해주세요. | | | | | | |
| ***오늘 의사는...*** | **아니다** | **보통**  **이다** | **그런 편**  **이다** | **그렇다** | **매우**  **그렇다** | **해당**  **없음** |
| **1. 의료진은 나를 편하게 대해주었다.**  (의료진은 나를 친근하고 따뜻하게 대했으며 존중해주었다. 차갑거나 퉁명스럽게 대하지 않았다.) |  |  |  |  |  |  |
| **2. 의료진은 내가 나의 이야기를 할 수 있게 해주었다.**  (의료진은 내가 나 자신의 말로 나의 병에 대해서 충분히 설명할 수 있는 시간을 주었다. 끼어들거나 관심을 다른 데로 돌리지 않았다.) |  |  |  |  |  |  |
| **3. 의료진은 내 말을 들어주었다.**  (의료진은 내가 하는 말에 유심히 주의를 기울이며 들었다. 내가 말하는 동안 컴퓨터나 차트만 보지 않았다) |  |  |  |  |  |  |
| **4. 의료진은 나를 전인적인 한 인간으로서 관심을 가지고 대했다.**  (의료진은 나의 생활과 상황에 대해 구체적으로 물었다/알고자 했다. 나를 환자 중 한명으로만 대하지 않았다.) |  |  |  |  |  |  |
| **5. 의료진은 나의 걱정을 충분히 이해하였다.**  (의료진은 나의 걱정을 정확히 이해하고 있음을 표현해 주었다. 나의 어떤 염려도 흘려듣거나 무시하지 않았다.) |  |  |  |  |  |  |
| **6. 의료진은 나에게 마음을 쓰며 공감을 보였다.**  (의료진은 나를 인간 대 인간으로 진심으로 염려하는 것처럼 보였다. 무관심하거나 거리를 두려고 하지 않았다) |  |  |  |  |  |  |
| **7. 의료진은 긍정적이었다.**  (의료진은 나의 문제를 긍정적으로 접근하고 긍정적 태도를 보였다. 나의 문제에 대해 솔직하지만 부정적이지 않았다.) |  |  |  |  |  |  |
| **8. 의료진은 나에게 분명하게 설명하였다.**  (의료진은 나의 질문에 충분히 대답해주었으며, 분명하게 설명하고, 알맞은 정보를 주었다. 모호하지 않았다.) |  |  |  |  |  |  |
| **9. 의료진은 내가 주도하도록 도와주었다.**  (의료진은 내가 스스로 건강을 향상시키기 위해 무엇을 할 수 있는지 함께 찾아봐 주었다. 나를 가르치기보다 북돋아주었다.) |  |  |  |  |  |  |
| **10. 의료진은 나와 함께 실행계획을 세웠다.**  (의료진은 내가 선택할 수 있는 것들에 대해 의논하고, 내가 원하는 만큼 결정에 참여할 수 있도록 하였다. 나의 입장을 무시하지 않았다.) |  |  |  |  |  |  |
| *Consultational and Relational Empathy CARE © Stewart W Mercer 2004, Korean version 2021* | | | | | | |
